# Analysis of Trend of Malaria Prevalence in the Ten Asian Countries from 2006 to 2011: A Longitudinal Study

**DOI:** 10.1155/2015/620598

**Published:** 2015-11-29

**Authors:** Shongkour Roy, Tanjina Khatun

**Affiliations:** ^1^Population Council, House No. 15B, Road No. 13, Gulshan, Dhaka 1212, Bangladesh; ^2^Department of Social Work, Masters Student, Government Bangla College, Mirpur 1, Dhaka 1216, Bangladesh

## Abstract

*Background.* To control the malaria mortality, the global and national communities have worked together and produced impressive results in the world. Some of the Asian counties' malaria mortality rate is more compared to countries with high health facilities around the world. This paper's main aim is to describe trend of malaria cases and mortality in 10 Asian countries using the World Health Organization data.* Methods.* Malaria mortality data was collected systematically from WHO and UN database for the period 2006–2011. We estimated malaria mortality by age and countries. We also explored the dynamic relationships among malaria death rate, total populations, and geographical region using a map. During 2006–2011, the average malaria death per 10,000 population of all ages was 0.239 (95% CI 0.104 to 0.373), of children aged less than 5 year 1.143 (0.598 to 1.687), and of age greater than 5 years 0.089 (0.043 to 0.137) in Asian countries. Malaria prevalence per 10,000 populations steadily decreased from 486.7 in 2006 to 298.9 in 2011.* Conclusion.* The findings show that malaria mortality is higher for children aged less than 5 years compared with with adults selected in Asian countries except Sri Lanka.

## 1. Introduction

Since the 1950s, the global health community has focused on eradication campaign to eliminate malaria and malaria related death all over the world [1]. But it failed globally because of some problems including the lack of innovative research and leaderships, the resistance of mosquitoes to insecticides used to kill them, the resistance of malaria parasites to drugs used to treat them, and implementation difficulties. The priority accorded to reductions of malaria mortality in developing countries is shown by its choice as the fourth Millennium Development Goals (MDGs). Much of recent malaria related studies have focused on infant mortality [2] and young children's and adult's exposure to the disease [3] and to some extent the impact on pregnant women [4], without classifying the malaria situation of other subgroups population. Scanty studies have looked specifically at the effect of malaria on adult and old-age people [5] and mobile group population [6]. A lot of data have been taken from heath facilities at different levels that may be dominated by patterns of health services utilization rather than clearly representing malaria patterns within communities [7]. Some works have taken any forms of data which are perhaps available and built to generalize patterns of malaria burden using proper statistical modeling techniques [8] and others have in-depth network members who maintain population surveillance in Health and Demographic Surveillance System sites across Africa and Asia [9].

Despite these efforts and visibility, there was broad concern that malaria remains a major cause of death in Asia [10] and much of infectious morbidity and 90% of malaria mortality are considered to be in many parts of Africa [11]. Although a lot of tools have been developed for malaria control in Asia, it has been argued that the enormous disease burden due to malaria is grossly underestimated [12]. In 2011, about 1.33 billion people or 75% of the region's total population resided in areas that were at risk of malaria in Asia. Among them there were 2144849 confirmed malaria cases and 1819 malaria deaths reported by the national malaria control programmes in Asia [13]. Malaria control and elimination in Southeast Asia reported that the 11 member countries in the WHO Southeast Asia region and among them 10 are endemic to malaria. Maldives has been malaria-free since 1984 [14] in south Asia.

In Asia, there have been significant malaria control efforts in recent years but it is unclear what impact they have had and six countries, Bhutan, Democratic People's Republic of Korea, Indonesia, Nepal, Sri Lanka, and Thailand, are aiming for malaria elimination as a longer-term goal. Sri Lanka is already in the elimination phase [15] and subnational malaria elimination is progressing well in Indonesia and Thailand [16]. This paper provides estimated malaria prevalence and mortality by age and country for 2006 to 2011. In addition, it allows us to produce maps of all ages' group mortality rate adjusted with total population.

## 2. Materials and Methods 

For the analysis subjects of this study we cover 10 countries out of 50 Asian counties. These countries have the highest malaria prevalence rate and close geographical region and fought against malaria for several decades. In this paper, to address the objective, our main aim is to estimate malaria mortality rate and its trend in countries over the said time period. The main sources of the longitudinal data are the World Health Organization and UN database. We utilize two sources (Table 1) of data to create trend of falciparum malaria mortality children under 5 years, children greater than 5 years, and all ages group population over the period 2006–2011 in Asian countries. All data has been publically available. The first task was the calculation of malaria mortality in Asian countries by age. The WHO database provides this malaria related death data by country and year. However, since some of this data was missing, we also created a second task to determine malaria mortality in Asian countries using the United Nation database. If a country was missing a value for malaria mortality in the WHO data, in that case, we replace that value of malaria mortality with the one from the UN data. The total population in million was collected from the World Development Indicator database. WHO and UN database provide good quality data; in previous studies many authors used multiple data sources that we also used in this study.

We analyzed descriptive statistics and trend of malaria mortality. The descriptive statistics are prevalence rate per 10,000 populations and death rate per 10,000 populations for all age. Prevalence rate is calculated using the number of malaria reported cases divided by total population with multiplying 10,000 populations for each country and year. Similarly, we also determined a death rate per 10,000 populations for all ages using the number of malaria attributed death divided by total population with multiplying 10,000 populations for each country and year.

In view of the differential patterns of malaria mortality, we analyzed the data into three age groups: aged under five years, aged greater than five years, and all ages using Stata software version 12.0 (StataCorp LP, Lakeway Drive College Station, Texas, USA). To identify summary measures of malaria related death in each country we estimated mean and standard deviation with confidence interval at 95% level of significance. The confidence interval indicates that there is a 95% probability of malaria death that encompasses the true values mean of malaria mortality at different age groups. We also exported the maps to view the geostatistical relationship between death rate for all ages and total population within countries.

## 3. Results

During 2006–2011, the prevalence rate per 10,000 people for Bangladesh was raised from 2.308 to 3.440, Myanmar from 43.573 to 96.261, Republic of Korea from 2.684 to 3.140, and Timor-Leste from 373.566 to 167.866. Again for Bhutan it decreased from 27.687 to 2.628, India from 15.428 to 10.555, Indonesia from 15.118 to 10.589, Nepal from 1.563 to 0.751, Sri Lanka from 0.298 to 0.059, and Thailand from 4.503 to 3.581 (Table 2). Compared to these major Asian countries, Myanmar had one of the highest prevalence rate of increase in malaria related to prevalence rate within this time period (Table 2).

The death rate per 10,000 people for India increased from 0.068 to 0.069 and for Indonesia from 0.100 to 0.123. On the other hand death rate for Bangladesh is decreased from 0.120 to 0.055, Bhutan from 0.133 to 0.014, Myanmar from 0.531 to 0.288, Nepal from 0.024 to 0.005, Sri Lanka from 0.001 to 0.000, Thailand from 0.023 to 0.012, and Timor-Leste from 2.642 to 0.935. The effective death rate change has been conducted in Sri Lanka and Republic of Korea within this time period in selected Asian countries.

**Figure 1:**
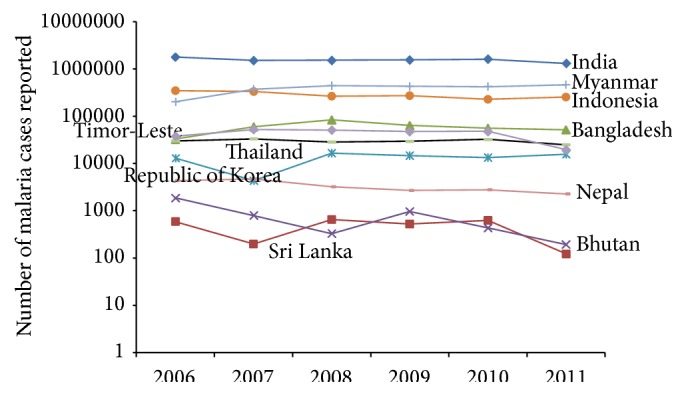
Trend of malaria reported cases (all ages) in Asian countries over 2006–2011.

**Figure 2:**
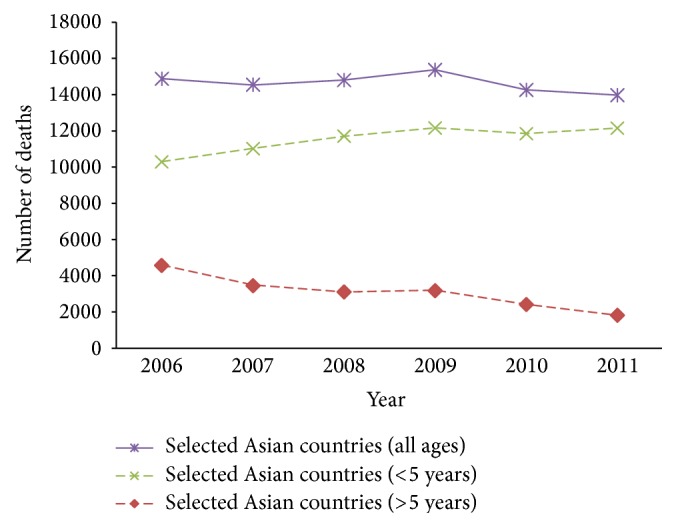
Trend of malaria deaths in geographical region over 2006–2011.

The malaria reported cases steadily changed in India and notably changed in Sri Lanka (Figure 1) and the number of malaria deaths was decreasing in both children aged greater than 5 and all ages but increasing in younger children aged less than 5 years (Figure 2). The overall rate of malaria prevalence decreased from 486.7 in 2006 to 298.8 in 2011 and death rate also decreased from 3.6 in 2006 to 1.5 in 2011 (Table 2) in Asia. Among ten Asian countries over the time period, the prevalence rate per 10,000 populations is still notably present in Republic of Korea and Sri Lanka but these countries have zero deaths.

**Figure 3:**
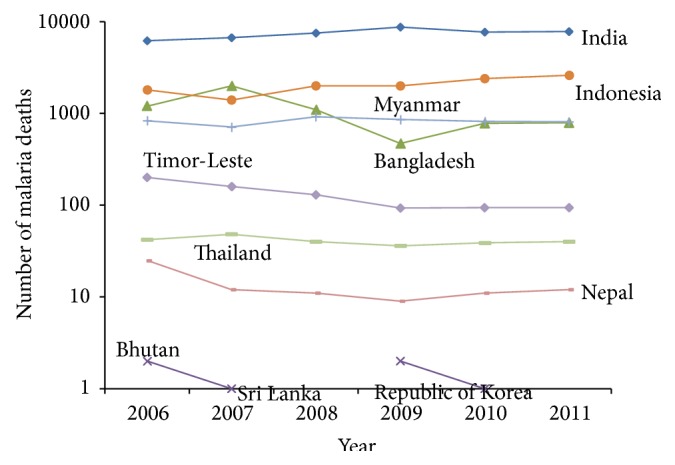
Trend of malaria reported death (aged < 5 year) in countries over 2006–2011.

**Figure 4:**
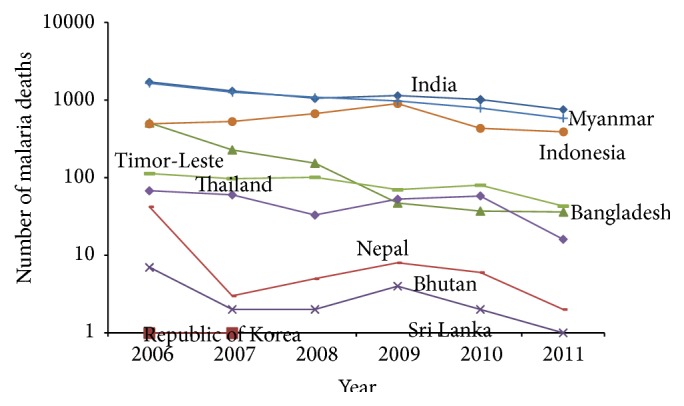
Trend of malaria reported death (aged > 5 year) in countries over 2006–2011.

Figure 3 showed the trend of number of malaria deaths under the age of 5 with zero deaths in Sri Lanka and Republic of Korea and consistent decrease in Nepal, Thailand, and also Timor-Leste. On the other hand, other selected countries followed an upward trend. When we considered malaria death age greater than 5, different pictures showed that zero deaths in Sri Lanka and Republic of Korea and others countries have a decreased trend over the period (Figure 4).

**Figure 5:**
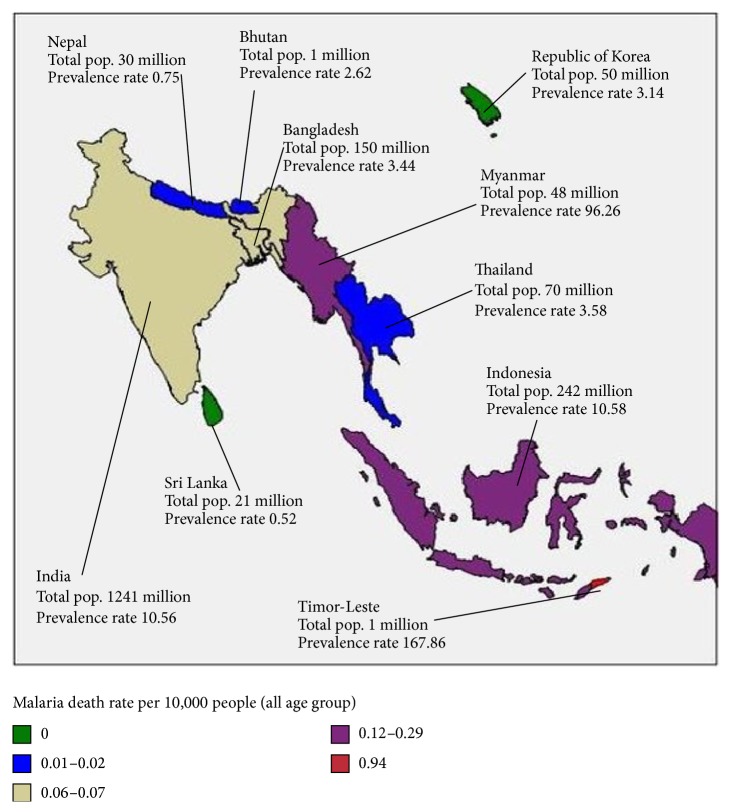
Total population and death rate per 10,000 population (all ages), 2011.

Between 2006 and 2011, the highest average malaria death rate in younger children aged less than 5 years of 6.867 (95% CI 4.767–8.969), children aged greater than 5 years of 0.539 (95% CI 0.286–0.793), and all ages of 1.639 (95% CI 0.987–2.292) was in Timor-Leste and the lowest was in Korea Republic (Table 3). Similarly, the highest malaria death rate per 10,000 population in Timor-Leste was 0.935 and in Republic of Korea was zero (Table 2, Figure 5) in 2011. The number of children deaths due to malaria in Asia has been steadily decreasing since 2009 (Figure 2). Contrary to this trend, the number of deaths due to malaria in Asian individuals aged greater than 5 years accounts for a significant decrease (Figure 2). The second highest average malaria death rate in younger children aged less than 5 years of 2.072 (95% CI 1.885–2.259) was in Myanmar and the third highest of 0.929 (95% CI 0.733–1.125) was in Indonesia (Table 3). But the third highest average malaria death rate in children aged greater than 5 years of 0.048 (95% CI 0.009–0.086) was in Bhutan (Table 3). When we looked at malaria death rate for the all age's group people in Table 3, the patterns that arose for the first, second, and third highest countries average malaria related deaths rate are Timor-Leste 1.639 (95% CI 0.987–2.292), Myanmar 0.397 (95% CI 0.309–0.486), and Indonesia 0.109 (95% CI12 0.093–0.126).

## 4. Discussion

Findings from analysis showed that, in the year 2011, on the basis of data pattern and a refined understanding about malaria mortality, malaria is the underlying cause of death for 13970 individuals, including 12150 children aged younger than 5 years and 1820 individuals aged 5 years or older. Since 2006, we noted that substantial improvement has been made in the combat against malaria, with a 6% reduction for all ages of malaria deaths in Asia, which was very low with global malaria death reduction rate [17]. However, our findings also show that the substantial acceleration in the decreases of death for ages of 5 years or older is 60% especially in Asia and provide hope that rapid reduction will be held in near future. The rate of increase of malaria death in children aged younger than 5 years is 18% since 2006. This rate of change in malaria mortality of children aged younger than 5 in Asian countries has become greater with previous estimates and does not support ambitious aspirational goals. Malaria mortality rate under 5 aged groups is still high compared to other aged groups in all most selected countries. Malaria death of children younger than 5 years is a serious issue in Asian countries, although a lot of initiatives have been taken to reduce malaria mortality. As new Asian aims for malaria mortality are decreased, it will be necessary to take lessons from these insights but also begin future planning for the evolving and more effective malaria reduction programme. Some achievement of random goals established without exact regard to the distribution of rates of change dominating at the time is a political form that confuses knowledge and acclaim for the substantial progress that has been made to reduce malaria mortality in the past decade. Moreover, accelerated decreases in malaria mortality will be more likely if we strongly maintain commitment for malaria control (zero death) and increase investment for new strategies, and evidence from policy responses in the Asian countries is widely and effectively circulated and implemented. Our future aimed to identify which factors have impact to reduce malaria mortality rate in Asian countries with included wide range of significant indicators.

## 5. Conclusions

The malaria mortality rate is steadily decreasing; moreover malaria is still now a major health hazard in Asia. Malaria prevalence rate in most of the selected Asian countries is not decreasing sharply as we expected and more attention is needed in these countries to control new cases of malaria. Our findings provided potential evidence that these countries' death rate is not increasing compared to increased prevalence rate. Our analysis refined that the children aged less than 5 were more affected by the malaria compared to older ones in Asian countries. Henceforth, children aged less than 5 are more vulnerable than others and scientific community also needs the highest priority to pay attention to this group of population.

## Figures and Tables

**Table 1:** Variables with measures and data sources.

Variables with measures	Sources
Malaria reported case (number)	World Data (World Bank, 2011)
Malaria death in children aged less than 5 years (number)	UN Data (2011)
Malaria death in children aged greater than 5 years (number)	UN Data (2011)
Malaria death in all ages group (number)	UN Data (2011)
Total population aged less than 5 years (number)	World Bank, 2011 (HNPS)
Total population aged greater than 5 years (number)	World Bank, 2011 (HNPS)
Total population (number)	WDI (2011)

**Table 2:** Country specific malaria prevalence rate per 10,000 people and death rate per 10,000 people (all ages).

Country(s)	Indicator(s)	Year(s)					
		2006	2007	2008	2009	2010	2011
Bangladesh	Prevalence	2.308	4.159	5.821	4.344	3.758	3.440
	Death rate	0.120	0.155	0.086	0.035	0.055	0.055

Bhutan	Prevalence	27.687	11.516	4.691	13.620	6.006	2.628
	Death rate	0.133	0.044	0.029	0.084	0.041	0.014

India	Prevalence	15.428	12.853	12.869	12.946	13.065	10.555
	Death rate	0.068	0.068	0.072	0.082	0.071	0.069

Indonesia	Prevalence	15.118	14.359	11.333	11.477	9.581	10.589
	Death rate	0.100	0.083	0.114	0.122	0.118	0.123

Myanmar	Prevalence	43.573	79.708	94.618	91.608	87.736	96.261
	Death rate	0.531	0.420	0.425	0.385	0.335	0.288

Nepal	Prevalence	1.563	1.671	1.117	0.926	0.932	0.751
	Death rate	0.024	0.005	0.006	0.006	0.006	0.005

Republic of Korea	Prevalence	2.684	0.894	3.394	2.975	2.711	3.140
	Death rate	0.000	0.000	0.000	0.000	0.000	0.000

Sri Lanka	Prevalence	0.298	0.099	0.321	0.260	0.306	0.059
	Death rate	0.001	0.000	0.000	0.000	0.000	0.000

Thailand	Prevalence	4.503	4.894	4.185	4.288	4.699	3.581
	Death rate	0.023	0.021	0.021	0.015	0.017	0.012

Timor-Leste	Prevalence	373.566	504.890	475.549	428.144	421.330	167.866
	Death rate	2.642	2.103	1.512	1.315	1.330	0.935

Ten Asian countries	Prevalence	486.728	635.043	613.898	570.589	550.123	298.970
	Death rate	3.642	2.900	2.263	2.044	1.974	1.500

**Table 3:** Summary statistics of country specific malaria mortality rate per 10,000 populations (2006–2011).

Country(s)	Malaria death (aged <5 year)				Malaria death (aged >5 year)				Malaria death (all ages)			
	Mean	s.d.	95% CI	Number of total pop.	Mean	s.d.	95% CI	Number of total pop.	Mean	s.d.	95% CI	Number of total pop.
Bangladesh	0.705	0.149	(0.321 to 1.089)	90675525	0.013	0.006	(−0.002 to 0.028)	787424472	0.084	0.019	(0.036 to 0.132)	878099997
Bhutan	0.139	0.051	(0.009 to 0.269)	428972	0.048	0.015	(0.009 to 0.086)	3813525	0.057	0.018	(0.011 to 0.104)	4242497
India	0.583	0.028	(0.512 to 0.655)	764549118	0.011	0.001	(0.007 to 0.014)	6431171424	0.072	0.002	(0.066 to 0.077)	7195720542
Indonesia	0.929	0.076	(0.733 to 1.125)	131110094	0.027	0.004	(0.017 to 0.036)	1285832423	0.109	0.006	(0.093 to 0.126)	1416942517
Myanmar	2.072	0.073	(1.885 to 2.259)	23892037	0.244	0.037	(0.150 to 0.338)	260780532	0.397	0.034	(0.309 to 0.486)	284672569
Nepal	0.037	0.007	(0.019–0.055)	21495313	0.004	0.003	(−0.002 to 0.011)	153495452	0.009	0.003	(0.00 to 0.017)	174990765
Republic of Korea	0	0	—	14392334	0	0	—	279897664	0	0	—	294289998
Sri Lanka	0	0	—	11056210	0.0002	0.0001	(−0.0002 to 0.0004)	111029790	0.0002	0.0001	(−0.0002 to 0.0004)	122086000
Thailand	0.091	0.003	(0.083 to 0.099)	26898214	0.013	0.002	(0.009 to 0.017)	383789512	0.018	0.002	(0.014 to 0.022)	410687726
Timor-Leste	6.867	0.817	(4.767 to 8.969)	1111807	0.539	0.099	(0.286 to 0.793)	5455026	1.639	0.254	(0.987 to 2.292)	6566833
Ten Asian countries	**1.143**	**0.272**	**(0.598 to 1.687)**	**1085609624**	**0.089**	**0.024**	**(0.043 to 0.137)**	**9702689820**	**0.239**	**0.066**	**(0.104 to 0.373)**	**10788299444**
